# Unsupervised machine learning improves risk stratification in newly diagnosed multiple myeloma: an analysis of the Spanish Myeloma Group

**DOI:** 10.1038/s41408-022-00647-z

**Published:** 2022-04-25

**Authors:** Adrian Mosquera Orgueira, Marta Sonia González Pérez, Jose Diaz Arias, Laura Rosiñol, Albert Oriol, Ana Isabel Teruel, Joaquin Martinez Lopez, Luis Palomera, Miguel Granell, Maria Jesus Blanchard, Javier de la Rubia, Ana López de la Guia, Rafael Rios, Anna Sureda, Miguel Teodoro Hernandez, Enrique Bengoechea, María José Calasanz, Norma Gutierrez, Maria Luis Martin, Joan Blade, Juan-Jose Lahuerta, Jesús San Miguel, Maria Victoria Mateos, the PETHEMA/GEM Cooperative Group, Adrian Mosquera Orgueira, Marta Sonia González Pérez, Jose Diaz Arias, Laura Rosiñol, Albert Oriol, Ana Isabel Teruel, Joaquin Martinez Lopez, Luis Palomera, Miguel Granell, Maria Jesus Blanchard, Javier de la Rubia, Ana López de la Guia, Rafael Rios, Anna Sureda, Miguel Teodoro Hernandez, Enrique Bengoechea, María José Calasanz, Norma Gutierrez, Maria Luis Martin, Joan Blade, Juan-Jose Lahuerta, Jesús San Miguel, Maria Victoria Mateos

**Affiliations:** 1 Hospital Clínico Universitario Santiago de Compostela, A Coruña, Spain; 2 Hospital Clínic, Institut d’investigacions Biomèdiques August Pi i Sunyer, Barcelona, Spain; 3 Institut Català d’Oncologia I Institut Josep Carreras, Hospital Germans Trias i Pujol, Badalona, Spain; 4 Hospital Clínico de Valencia, Valencia, Spain; 5 Hospital Universitario 12 de Octubre, i+12, Complutense University, CNIO, Madrid, Spain; 6 Hospital Clínico Lozano Blesa, Zaragoza, Spain; 7 Hospital Sant Pau, Barcelona, Spain; 8 Hospital Ramón y Cajal, Madrid, Spain; 9 Hospital Doctor Peset, Valencia, Spain; 10 Hospital Universitario La Paz, Madrid, Spain; 11 Hospital Virgen de las Nieves, CIBERESP, Ibs, Granada, Spain; 12 Institut Català d’Oncologia-Hospitalet, IDIBELL, Universitat de Barcelona, Barcelona, Spain; 13 Hospital Universitario de Canarias, Santa Cruz de Tenerife, Spain; 14 Hospital de Donostia, San Sebastian, Spain; 15 Clínica Universidad de Navarra, CIMA, CIBERONC, IDISNA, Pamplona, Spain; 16 Hospital Universitario de Salamanca, Instituto de Investigación Biomédica de Salamanca, Instituto de Biología Molecular y Celular del Cáncer (Universidad de Salamanca-Consejo Superior de Investigaciones Científicas), CIBERONC, Salamanca, Spain; 17 Hospital Clínico Universitario Santiago de Compostela, A Coruña, Spain; 18 Hospital Clínic, Institut d’investigacions Biomèdiques August Pi i Sunyer, Barcelona, Spain; 19 Institut Català d’Oncologia I Institut Josep Carreras, Hospital Germans Trias i Pujol, Badalona, Spain; 20 Hospital Clínico de Valencia, Valencia, Spain; 21 Hospital Universitario 12 de Octubre, i+12, Complutense University, CNIO, Madrid, Spain; 22 Hospital Clínico Lozano Blesa, Zaragoza, Spain; 23 Hospital Sant Pau, Barcelona, Spain; 24 Hospital Ramón y Cajal, Madrid, Spain; 25 Hospital Doctor Peset, Valencia, Spain; 26 Hospital Universitario La Paz, Madrid, Spain; 27 Hospital Virgen de las Nieves, CIBERESP, Ibs, Granada, Spain; 28 Institut Català d’Oncologia-Hospitalet, IDIBELL, Universitat de Barcelona, Barcelona, Spain; 29 Hospital Universitario de Canarias, Santa Cruz de Tenerife, Spain; 30 Hospital de Donostia, San Sebastian, Spain; 31 Clínica Universidad de Navarra, CIMA, CIBERONC, IDISNA, Pamplona, Spain; 32 Hospital Universitario de Salamanca, Instituto de Investigación Biomédica de Salamanca, Instituto de Biología Molecular y Celular del Cáncer (Universidad de Salamanca-Consejo Superior de Investigaciones Científicas), CIBERONC, Salamanca, Spain

**Keywords:** Risk factors, Myeloma

## Abstract

The International Staging System (ISS) and the Revised International Staging System (R-ISS) are commonly used prognostic scores in multiple myeloma (MM). These methods have significant gaps, particularly among intermediate-risk groups. The aim of this study was to improve risk stratification in newly diagnosed MM patients using data from three different trials developed by the Spanish Myeloma Group. For this, we applied an unsupervised machine learning clusterization technique on a set of clinical, biochemical and cytogenetic variables, and we identified two novel clusters of patients with significantly different survival. The prognostic precision of this clusterization was superior to those of ISS and R-ISS scores, and appeared to be particularly useful to improve risk stratification among R-ISS 2 patients. Additionally, patients assigned to the low-risk cluster in the GEM05 over 65 years trial had a significant survival benefit when treated with VMP as compared with VTD. In conclusion, we describe a simple prognostic model for newly diagnosed MM whose predictions are independent of the ISS and R-ISS scores. Notably, the model is particularly useful in order to re-classify R-ISS score 2 patients in 2 different prognostic subgroups. The combination of ISS, R-ISS and unsupervised machine learning clusterization brings a promising approximation to improve MM risk stratification.

## Introduction

The International Staging System (ISS) has been the most used prognostic score employed for risk stratification in newly diagnosed Multiple Myeloma (MM) patients. This score is based on surrogate markers of myeloma cell biology and host factors: ß2-microglobulin and albumin [1]. The ISS stratifies patients in three subgroups with an overall survival (OS) of 62, 44, and 22 months respectively, and it has been validated in several studies and clinical trials. The main limitation of this model is that it does not incorporate any genetic or proliferation biomarkers of the disease.

A revision of the ISS was presented in 2015 which incorporated elevated lactate dehydrogenase (LDH) plus t(4;14), t(16;14) & del(17p) as high-risk cytogenetics abnormalities [2]. This score identified 3 risk groups with a median OS of 43, 83 months and not reached. Only 10% of patients were allocated to the high-risk group (R-ISS 3), 28% were assigned to the low-risk group (R-ISS 1) and most patients (62%) were classified as intermediate risk (R-ISS 2). It has become progressively evident that some patients who belong to the R-ISS 1 low-risk group have poor survival, whereas the outcome of patients in the intermediate group (R-ISS 2) is very heterogeneous. Additionally, recent reports highlight that both ISS and R-ISS have similar predictive performance, suggesting that optimized data exploitation tools might help to bring improved risk stratification techniques to the field [3]. All these issues highlight the limitations of these scores, which fail to properly stratify many patients.

Survival prediction of patients with hematological cancer has been extensively improved in the last years. For example, several biomarker panels based on next-generation sequencing of recurrently mutated or aberrantly expressed genes have been proposed to facilitate prognostic stratification in acute myeloid leukemia, myelodysplastic syndromes and lymphomas, and indeed various studies have proved that these novel personalized models fitted with machine learning algorithms outperform the precision of currently established prognostic tools [4–6]. More recently, other sophisticated risk stratification methods, using gene expression profiling, comprehensive cytogenetic assessments or next generation sequencing strategies have been published, but in clinical practice, these are rarely employed due to the lack of availability, high cost as well as technical and logistical difficulties [7–9].

Therefore, improved risk stratification of MM with ready-to-use information is much awaited. In this line, the recent development of machine learning in medicine has become key to overcome some of the limitations of classical prognostic scores. Machine learning is a field of artificial intelligence where prediction is based on the modeling of outcomes considering complex interactions between multiple variables derived from real examples, rather than on the application of human-made rules. In the particular case of MM, such advanced techniques can optimize the number of prognostic groups and the assignment of patients to these according to flexible data structures, instead of the rigid thresholds implemented in the current clinical scores. With this in mind, we have developed a new unsupervised machine learning model for MM risk stratification by integrating clinical, biochemical and cytogenetic data at diagnosis through the use of datasets corresponding to series of MM patients homogeneously treated in the context of clinical trials conducted by the Spanish Myeloma Group. Our results indicate that this strategy can significantly improve MM prognostication, particularly among patients assigned to the R-ISS 2 intermediate-risk group.

## Materials and methods

### Data source

We retrieved original data from three clinical trials developed by the Spanish Myeloma Group (*Grupo Español de Mieloma*, GEM), namely GEM05 under 65 years [10], GEM05 over 65 years [11] and GEM2012 under 65 years [12]. All trials evaluated different upfront treatments in newly diagnosed MM.

Patients included in the GEM05 under 65 years trial were randomized (1:1:1) to receive 4 alternating cycles of vincristine, BCNU, cyclophosphamide, melphalan and prednisone (VBMCP) - vincristine, BCNU, adriamycin, dexamethasone (VBAD) + 2 cycles of bortezomib (Group A) or 6 cycles of thalidomide + dexamethasone (TD) (Group B) or thalidomide + dexamethasone + bortezomib (VTD) during 24 weeks (Group C). Eligible patients underwent autologous stem cell transplantation, and 3 months after transplant patients were randomized to three different maintenance arms: either Interferon a-2b, thalidomide or thalidomide plus bortezomib for 2 years.

Patients included in the GEM05 over 65 years trial were randomized 1:1 to receive melphalan + prednisone + bortezomib (VMP, Group A) or thalidomide + prednisone + bortezomib (VTD, Group B). All patients received induction treatment for up to 30 weeks. Patients were further randomized 1:1 to receive maintenance treatment: either Thalidomide + Bortezomib (Group M1) or Prednisone + Bortezomib (Group M2) for three years after four weeks if no progression or toxicity.

Patients included in the GEM2012 under 65 years trial were treated with six cycles of induction treatment with bortezomib + lenalidomide + dexamethasone (VRD). After induction, patients were randomized 1:1 to receive an autologous transplant with melphalan 200 mg/m2 (MEL200) versus Busulfan 12 mg/kg plus melphalan 140 mg/m2 (BUMEL) as conditioning regimens. Three months after transplantation, patients received two cycles of consolidation treatment with VRD at the same doses administered during induction treatment. Those patients in response after two cycles of consolidation therapy with VRD were further included in a second maintenance trial (GEM MAIN 2014), being randomized to lenalidomide +/− ixazomib for 2–5 years depending on MRD analysis.

Cytogenetic analysis was performed using fluorescence in situ hybridization (FISH) on whole bone marrow (GEM05 trials) or CD138-selected plasma cells (GEM2012 trial), and included t(4;14), t(14;16) and 17p deletion in all trials. Among the remaining annotations, the following common baseline variables were retrieved: immunoglobulin light and heavy chain type, Durie-Salmon staging, monoclonal spike in blood and urine, hemoglobin, creatinine, albumin, albumin-adjusted calcium, ß2-microglobulin, elevated LDH and percentage of plasma cells in bone marrow aspirate smear. Note that in the case of Durie-Salmon annotation, both parts of the staging system were analyzed separately. In this regard, one variable analyzed the presence of kidney disfunction (Durie-Salmon stages A and B), whereas another variable reflected the classification of patients in 3 groups (Durie-Salmon stages I, II & III) based on hemoglobin, calcium, presence of bone X-ray abnormalities/plasmocitomes and monoclonal components in serum and urine. Patients who had incomplete annotation for any of the variables were discarded from downstream analysis.

Overall survival (OS) was defined as time from diagnosis to death from any cause, and progression-free survival (PFS) was defined as time from diagnosis to disease progression or death from any cause.

### Variable selection and model development

Statistical analysis was performed on R version 4.1.0 [13]. Survival analysis was performed with the “survival” package version 3.2.11 [14]. Univariate cox-regression was used to test the association of each variable with overall survival in the largest cohort (GEM05 under 65 years). Principal components were extracted using *Factor Analysis for Mixed Data* (FAMD) implemented in the *FactoMineR* version 2.4 package [15], which can accept continuous and categorical variables as input. In each case, we selected as many principal components as variables included in the model.

Unsupervised clustering was performed using Gaussian Mixture Modeling fitted with an Expectation Maximization algorithm (GMM-EM model implemented in the “Mclust” algorithm version 5.4.7) [16]. Briefly, the Mclust algorithm determines the most likely set of patient clusters according to geometric properties (distribution, volume, and shape). An EM algorithm is used for maximum likelihood estimation, and the best model is selected according to Bayes information criteria. Inferred clusters in the GEM05 under 65 years trial were used to predict clusterizations in the remaining datasets (GEM05 over 65 years and GEM2012 under 65 years). Cox regression was used to analyze the association of such clusters with OS and PFS, as well as their relationship with the International Staging System (ISS) and revised ISS (R-ISS) scores. Assumption of proportional hazards was tested with Schoenfeld’s method. Model’s precision was assessed using cross-validated cox models and time-dependent Area Under the Curve (AUC) were calculated at different time points with 500 bootstraps using the *riskRegression* package version 2021.10.10 [17]. Model’s discriminative power was assessed using 10-fold cross-validated Harrel’s concordance indexes (c-indexes) implemented in the *RMS* package version 6.2.0 [18]. Finally, survival curves were plotted using the Kaplan-Meier method.

## Results

### Variable selection and unsupervised model fitting

Baseline characteristics of the patients included in each cohort are represented in Table 1. The first analysis was done in the cohort of patients included in the GEM05 under 65 years trial, as this was the study with the largest number of patients with complete annotation data available. We identified 10, 14 and 16 variables which were associated with OS at *p* value thresholds of 0.01, 0.05 and 0.1 (Table 2). FAMD decomposition was performed taking as input all cytogenetic variables (17p deletion, t(4;14), t(14;16) and any high risk cytogenetic alteration) plus any of the remaining variables at p-value thresholds of 0.01, 0.05 and 0.1. Afterwards, GMM-EM was implemented to identify the optimal two clusters in the database (Table 3). Clusterization results were significantly associated with OS regardless of the *p* value threshold used, but statistical significance was superior with the *p* value threshold of 0.01 (*p* value 7.44 × 10^−8^, HR 0.35) compared with the *p* value thresholds of 0.05 (*p* value 1.63 × 10^−4^, HR 0.47) and 0.1 (*p* value 2.65 × 10^−5^, HR 0.42).

**Table 1:** Baseline characteristics of selected patients in the different trials.

	GEM05 under 65	GEM05 over 65	GEM2012 under 65
N	305	218	229
% High Risk Cytogenetics	19.34%	18.80%	26.63%
Durie-Salmon stages: I, II & III	6.23%, 48.85%, 44.92%	7.34%, 51.83%, 40.83%	10.48%, 38.43%, 51.09%
Durie-Salmon stages A & B	96.40%, 3.60%	95.87%, 4.13%	97.82%, 2.18%
ISS stages: I, II, III	38.36%, 41.31%, 20.33%	24.31%, 43,58%, 32.11%	43.23%, 30.57%, 26.20%
RISS stages: I, II, III	28.52%, 62.62%, 8.85%	19.72%, 70.64%, 9.63%	27.94%, 61.57%, 10.48%
Median serum monoclonal spike (g/dL)	3.9	3.6	2.8
Median urine monoclonal spike (g/dL)	0.19	0.20	0.14
Median hemoglobin (g/dL)	10.8	10.4	11.1
Median albumin-adjusted calcium (mg/dL)	9.68	9.95	9.58
Median B2-microglobulin (mg/dL)	3.3	4.0	3.4
Raised LDH	15.73%	12.84%	16.52%
Median plasma cells in bone marrow smear	36%	35%	28%
Presence of major myeloma-related skeletal injuries	33.44%	25.23%	35.81%
Presence of plasmocitomes	17.05%	13.30%	22.71%

**Table 2:** Cox regression testing the association of the 18 variables with overall survival in the GEM05 under 65 years cohort.

Variable	*p* value
High risk cytogenetics	5.36 × 10^−5^
t(14;16)	0.04
17p deletion	0.09
t(4;14)	1.91 × 10^−5^
Immunoglobulin subclass	0.51
Durie-Salmon stages (I, II & III)	1.86 × 10^−3^
Durie-Salmon stages (A & B)	0.21
Serum M spike	0.98
Urine M spike	0.15
Hemoglobine	2.06 × 10^−3^
Creatinine	0.80
Albumin	0.01
Albumin-adjusted calcium	4.49 × 10^−3^
B2-microglobulin	9.29 × 10^−8^
Raised LDH	1.75 × 10^−6^
% of bone marrow plasma cells	0.04

**Table 3:** Distribution of 2 clusters detected with unsupervised clustering across cohorts, as well as cox regression testing the association with overall survival and progression-free survival.

	GEM05 under 65	GEM05 over 65	GEM2012 under 65
% patients in each cluster	36.72%, 63.28%	34.86%, 65.13%	44.10%, 55.90%
*p* value OS	7.44 × 10^−8^	8.07 × 10^−5^	1.42 × 10^−3^
HR (95% CI) for OS	0.35 [0.24, 0.52]	0.51 [0.36, 0.71]	0.36 [0.19, 0.68]
*p* value PFS	2.48 × 10^−4^	1.16 × 10^−3^	5.47 × 10^−4^
HR (95% CI) for PFS	0.60 [0.45, 0.79]	0.60 [0.45, 0.82]	0.50 [0.34, 0.74]
*p* value OS (RISS adjusted)	0.01	9.85 × 10^−3^	0.02
HR (95% CI) for OS (RISS adjusted)	0.56 [0.36, 0.87]	0.591 [0.40 0.88]	0.42 [0.20, 0.89]
*p* value PFS (RISS adjusted)	0.15	0.12	0.02
HR (95% CI) (RISS adjusted)	0.79 [0.57, 1.09]	0.75 [0.53, 1.07]	0.57 [0.36, 0.92]
*p* value OS (ISS adjusted)	1.96 × 10^−5^	1.01 × 10^−3^	3.47 × 10^−3^
HR (95% CI) for OS (ISS adjusted)	0.42 [0.28, 0.63]	0.55 [0.39, 0.79]	0.39 [0.20, 0.73]
*p* value PFS (ISS adjusted)	5.37 × 10^−3^	0.01	1.24 × 10^−3^
HR (95% CI) (ISS adjusted)	0.66 [0.50, 0.89]	0.67 [0.49, 0.91]	0.52 [0.35, 0.77]

Aside from cytogenetic data, this optimal model included the following variables: Durie-Salmon staging (I, II and III), hemoglobin, albumin-adjusted calcium, ß2-microglobulin and elevated LDH. We also tested the prognostic impact of GMM-EM-based models with 3 and 4 optimal clusters using this set of variables, but results were inferior to the model based on 2 optimal clusters (Supplementary Fig. 1).

**Fig. 1:**
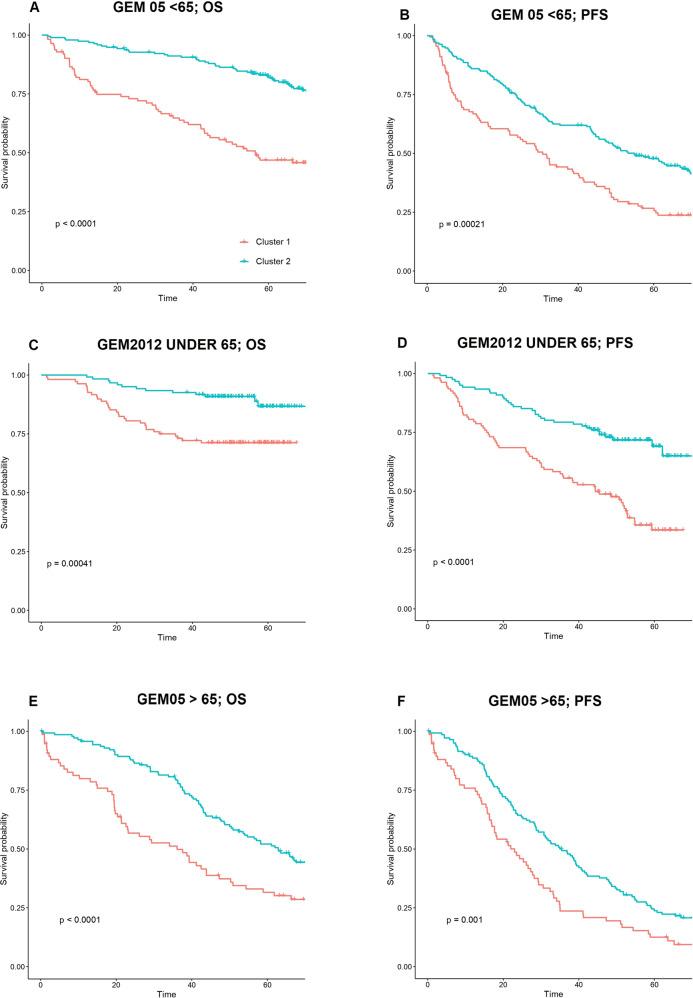
Patient outcomes according to the novel prognostic score. Kaplan–Meier curves representing the impact of the 2 clusters detected through unsupervised machine learning on overall survival (OS) and progression-free survival (PFS) in the 3 trial cohorts. “P” symbol indicates *p*-value. **A**, **B** OS and PFS for the GEM2005 under 65 years trial. **C**, **D** OS and PFS for the GEM2012 under 65 years trial. **E**, **F** OS and PFS for the GEM2005 over 65 years trial.

Predictions created by this model on GEM05 under 65 years were confirmed on GEM05 over 65 years and GEM2012 and two different clusters of patients were identified in both cases (Fig. 1). Additionally, this clusterization was significantly associated with OS in both cohorts (cox *p* value 1.42 × 10^−3^, HR 0.36 in the GEM2012 under 65 years cohort & cox *p* value 8.07 × 10^−5^, HR 0.51 in the GEM05 over 65 years cohort; Table 3).

Furthermore, the clusterization was significantly associated with PFS in all cohorts. (Fig. 1). The characteristics of cluster 1 and cluster 2 patients according to the constituting variables of the unsupervised model are represented in the Supplementary Table 1.

### Relationship of unsupervised clusterization with ISS and R-ISS

**Fig. 2:**
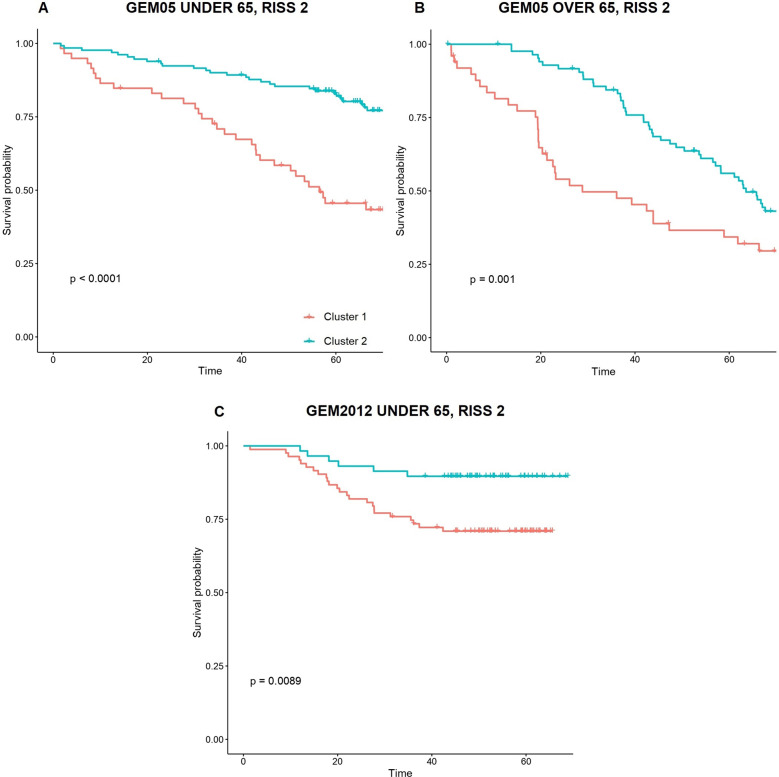
Survival of R-ISS 2 patients according to the new score. Impact of the 2 clusters detected with unsupervised machine learning on overall survival of R-ISS 2 MM patients across the 3 trial cohorts, namely GEM2005 under 65 years trial (**A**), GEM2005 over 65 years trial (**B**) and GEM2012 under 65 years trial (**C**).

**Fig. 3:**
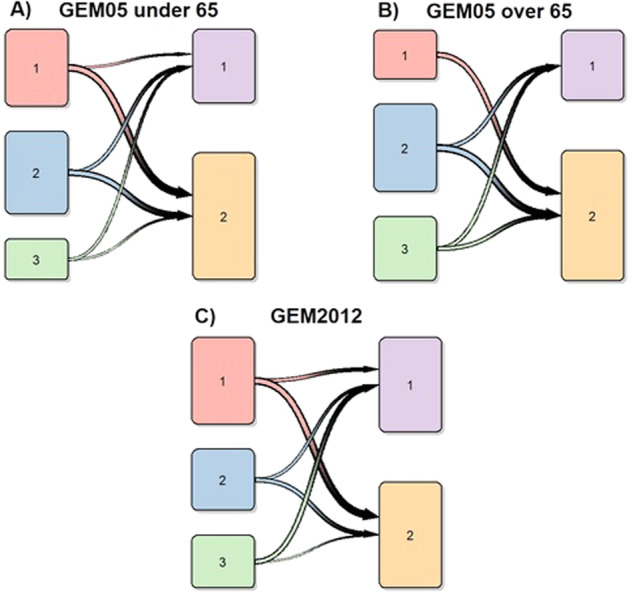
Transition plots between ISS scores and unsupervised risk clusters in the 3 different clinical trials evaluated. ISS scores are represented on the left column of each graph, and unsupervised clusters are represented on the right side. Transition plots for the GEM2005 under 65 years, GEM2005 over 65 years and GEM2012 under 65 years trials are represented in plots **A**, **B** and **C**, respectively.

The unsupervised clusterization model was associated with OS independently of ISS and R-ISS scores in all cohorts (multivariate cox *p* value <0.05, Table 3). Additionally, the clusterization was associated with PFS independently of ISS stages in all cases, although the GEM2012 under 65 years trial was the only population in which it was independent of R-ISS stage (Table 4). These findings motivated a subanalysis by ISS and R-ISS scores (Table 4, Supplementary Fig. 2 and 3). Interestingly, we observed that the unsupervised model was particularly useful to stratify patients with R-ISS 2 into two clusters with significantly divergent OS curves in all cohorts (Fig. 2). Indeed, only a minority of patients with either R-ISS 1 or R-ISS 3 were reclassified to a higher or lower risk classification, respectively: 1 R-ISS 3 patient was assigned to the lower risk cluster, and 6 R-ISS 1 patients were assigned to the higher risk cluster. On the contrary, remarkable changes in risk group assignment were observed between ISS scores and these new risk clusters (Fig. 3).

**Table 4:** Patient distribution according to ISS/R-ISS scores and unsupervised clustering results.

GEM05 under 65	Cluster 1	Cluster 2	Cluster 1 vs Cluster 2 *p* value
*ISS 1*	9.09%	29.18%	<1 × 10^−4^
*ISS 2*	15.74%	25.57%	0.02
*ISS 3*	11.80%	8.52%	0.21
*RISS 1*	0.98%	27.54%	0.49
*RISS 2*	26.88%	35.74%	6.10 × 10^−3^
*RISS 3*	8.85%	0%	NA

GEM05 over 65	Cluster 1	Cluster 2	Cluster 1 vs Cluster 2 OS *p* value
*ISS 1*	9.18%	28.18%	0.32
*ISS 2*	15.74%	25.57%	0.06
*ISS 3*	11.80%	8.52%	0.02
*RISS 1*	0.98%	27.54%	NA
*RISS 2*	26.89%	35.74%	8.20 × 10^−3^
*RISS 3*	8.85%	0%%	NA

GEM2012 under 65	Cluster 1	Cluster 2	Cluster 1 vs Cluster 2 OS *p* value
*ISS 1*	15.72%	27.51%	0.57
*ISS 2*	11.79%	18.78%	4.50 × 10^−3^
*ISS 3*	16.59%	9.61%	0.08
*RISS 1*	1.31%	26.63%	0.52
*RISS 2*	32.75%	28.82%	0.01
*RISS 3*	10.04%	0.44%	0.55

Statistical significance (cox *p* values) for differential OS between both clusters in each subgroup is shown.

On the other side, the distribution of ISS scores in patients assigned to both prognostic clusters was more heterogeneous, and our new clusterization tended to provide additional prognostic stratification in most cases (particularly in ISS 2 and 3 groups; Table 4).

### Survival analysis of R-ISS 2 subgroups

We analyzed the survival of the two new clusters of R-ISS 2 patients identified with this novel approach (low-risk and high-risk), and compared them with the survival of patients with R-ISS 1 and 3 MM, respectively (Supplementary Table 2, Supplementary Fig. 4). Interestingly, we found no significant differences in survival between these subgroups in the three different clinical trials evaluated, with the exception of a significant difference between R-ISS 1 and R-ISS 2 low-risk groups in the GEM05 under 65 years trial.

A focused analysis on R-ISS 2 patients evidenced that the key variables to stratify patients in two different risk clusters was the presence of high-risk cytogenetics or raised LDH, as all R-ISS 2 patients with any of these features were assigned to the higher-risk cluster I. However, a variable proportion of R-ISS 2 cluster I patients had standard-risk cytogenetics and normal baseline LDH. Importantly, their survival was similar to that of patients with high risk cytogenetics or raised LDH (Supplementary Table 3), reinforcing their membership to the higher risk cluster.

### Discriminative power and precision of the different risk stratification models

We used cox c-indexes to investigate the discriminative capacity of each score and score combination on the different cohorts (Table 5). R-ISS was superior to ISS only in GEM05 under 65 years, and both scores performed similarly in the remaining datasets. Additionally, our unsupervised clusterization model achieved superior c-indexes than ISS in all cohorts, superior c-indexes than R-ISS in the cohorts of patients included in the GEM05 over 65 years and GEM12 under 65 years, and similar concordance to R-ISS in the GEM05 under 65 years cohort. Importantly, the combination of ISS and R-ISS achieved inferior c-indexes than any of the combinations that included our unsupervised clusterization model. 10-fold cross-validation confirmed the robustness of the prognostic clusters (Supplementary Table 4).

**Table 5:** C-indexes and corresponding standard errors in cox regression including ISS scores, R-ISS scores and unsupervised clustering results.

	GEM05 under 65	GEM2012 under 65	GEM05 over 65
ISS	0.619 (0.026)	0.596 (0.039)	0.577 (0.022)
RISS	0.653 (0.02)	0.606 (0.033)	0.570 (0.02)
UNSUPERVISED MODEL	0.645 (0.023)	0.636 (0.035)	0.593 (0.021)
ISS + RISS	0.652 (0.024)	0.618 (0.038)	0.591 (0.024)
ISS + UNSUPERVISED MODEL	0.696 (0.023)	0.664 (0.04)	0.62 (0.025)
RISS + UNSUPERVISED MODEL	0.694 (0.023)	0.653 (0.038)	0.607 (0.024)
ISS + RISS + UNSUPERVISED MODEL	0.704 (0.023)	0.661 (0.04)	0.621 (0.025)

Univariate and multivariate cox regression models were fitted.

Time-dependent AUCs revealed that the precision in survival prediction of the unsupervised clusterization was clearly superior to ISS and R-ISS in most cases (Supplementary Fig. 5). In the case of GEM 2005 < 65 years, we observed a less superior performance when compared to the R-ISS. However, this cohort was used to develop the R-ISS score itself, so some degree of overfitting could exist [2].

### Relationship of unsupervised clusterization with treatment outcomes in the different clinical trials

**Fig. 4:**
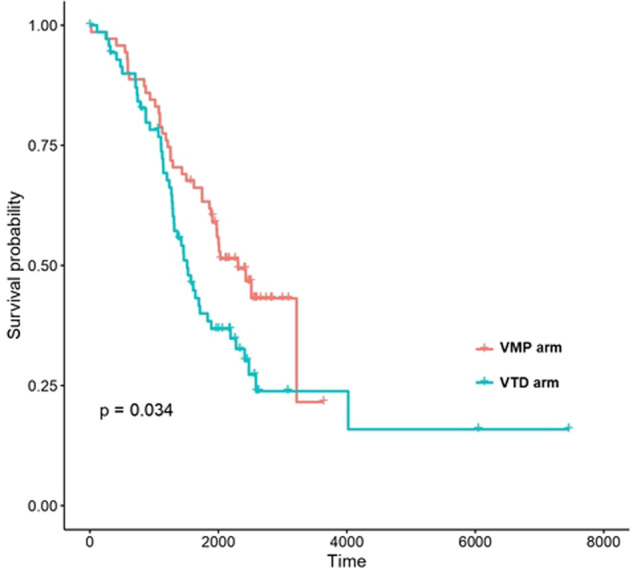
Impact of the new score system on drug response. Representation of overall survival curves of patients belonging to Cluster 2 treated with VMP or VTD.

In the GEM05 under 65 years trial, survival of both clusters of patients was similar regardless of the assignment to the different induction or post-transplant maintenance arms. Similarly, survival of the two clusters was similar regardless of the type of conditioning regime evaluated in the GEM2012 under 65 years trial. Finally, a benefit in terms of OS was identified for patients in the low-risk cluster when treated with VMP compared with VTD in the GEM05 over 65 years trial (*p* value 0.03, Fig. 4). However, no significant differences were observed between both clusters when considering the different maintenance strategies performed in this trial.

## Discussion

In the present work, we describe a new prognostic classification of newly diagnosed MM based on the application of intelligent information technologies to clinical trial data produced by the Spanish Myeloma Group. During the last 20 years, different prognostic models have been developed to stratify newly diagnosed MM patients, among which ISS and R-ISS are the most commonly used. Both models appear useful in identifying a small subgroup of high risk patients. However, the main limitation resides in the fact that most patients are categorized in low or intermediate categories. Unfortunately, some patients classified as low-risk (ISS 1 and R-ISS 1) have a short survival, whereas the majority of patients included in the largest R-ISS 2 group have unpredictable outcomes.Therefore, new approximations based on advanced data analytics are needed in the prognostic stratification of MM patients.

Several reasons may explain the limitations of these scores. For example, 19% of patients belonging to ISS 1 and 25% of patients in ISS 2 present high risk cytogenetic abnormalities [2]. In addition to that, the choice of cytogenetic abnormalities included in R-ISS may be suboptimal. Indeed, some authors have associated other cytogenetics abnormalities with long-term outcomes (e.g., 1p deletion and 1q amplification) [19–21]. Moreover, it has been reported that the weight of each cytogenetic alteration may be different (additive score) [22], and that some cytogenetic abnormalities can “modulate” the effect of others. In fact, a type of double hit myeloma with very poor prognosis has been recently described, which is characterized by either biallelic *TP53* inactivation or amplification (≥4 copies) of *CKS1B* (1q21) on the background of ISS III [23].

In an attempt to optimize prognostic scores, some groups have reported prognostic models based on genetic factors, using high-throughput genomic analysis that are more sophisticated than FISH [7–9]. Although these approaches seem very promising, they are not easily applicable in real life due to cost and technological complexity. Beyond genetic factors, there are other well-known prognostic factors such as renal failure, extramedullary disease or circulating plasma cells that are not openly present in either ISS nor R-ISS scores [24–26]. Notably, a large list of new prognostic factors (socioeconomic status, some comorbidities, frailty score, diagnostic delay, the specific type of myeloma defining event, immunoparesia…) or those with a lower level of evidence could also impact the outcome but they are not usually taken into account [27]. Furthermore, MM clinical evolution is recurrently impacted by the appearance of new drugs & drug combinations, and the quality of the response to these drugs is one of the most powerful prognostic factors. Although current models cannot be used to predict optimal schemes, some groups, including ours [28], are working in this area with encouraging results.

New information technologies, such as big data and machine learning algorithms, provide the opportunity to create more precise models in order to optimize risk stratification based on individual clinical and biological factors. Particularly, unsupervised machine learning algorithms (as the one used in this paper) come along with substantial benefits when identifying patient subgroups. Importantly, there is no prior assumption about cluster composition, as these are inferred from complex patterns in the data without the need to provide human-made instructions. Therefore, this strategy does not rely on simple optimal cut-offs, but can instead identify which is the most likely composition of patient clusters and improve the assignment of each patient even though he or she might be in the boundaries of the distributions. In this way, unsupervised machine learning strategies help researchers to maximize the value of the data by facilitating the conversion of multidimensional data into simplified, optimized and reproducible clusterizations. Using such an approach, we have created a simple and easy to use prognostic model based on 9 clinical and biological variables which arose from patients included in GEM05 under 65 years clinical trial. Although this trial included conventional chemotherapy and interferon as maintenance in one of its arms, we have subsequently validated the model in 2 other clinical trials which used new drugs (proteasome inhibitors and immunomodulatory drugs) with similar results. Importantly, all variables included in this model are readily available to any patient in clinical practice and there is no need for sophisticated technologies.

The unsupervised model identified 2 clusters of patients with different PFS and OS independently of ISS and R-ISS scores in all cohorts. More interestingly, our model was particularly useful to stratify patients with R-ISS score 2 into 2 clusters with significantly divergent OS curves in the 3 cohorts, and of note, all patients with high LDH or high-risk cytogenetics were assigned to the high risk cluster. To our knowledge, this is the first model that enables such differentiation. Importantly, the model retained its predictive power independently of induction type, transplantation conditioning and the different maintenance schemes. Additionally, time-dependent AUCs and c-indexes indicated that the new clusterization was superior to either ISS or R-ISS in most cases. Despite the fact that both the ISS and R-ISS share some variables with the new prognostic model, the latter provides additional discriminative value to the former two. Future efforts should pursue an optimal entangling of the variables included in this new model with those of the ISS and R-ISS, so as to achieve a single model on top of these that can integrate all the prognostic information into well-defined prognostic groups. Finally, the unsupervised clusterization model identified a subgroup of low risk patients who had longer OS when VMP was used as induction compared to VTD in the GEM05 over 65 years trial. Although this information might not be very relevant in clinical practice today because of new standards of care, it must be evaluated whether this model can help us to individualize the best option of therapy in the setting of the new standards of care.

This analysis emphasizes the importance of an optimal application of information technologies to patient data in order to improve disease prognostication. Even though machine learning models are frequently developed with big chunks of data which might hinder their broad applicability [29], our results indicate that it is possible to significantly improve disease prognostication by re-interpreting a limited number of classical variables. Therefore, relevant scientific advances might be achieved in similar scenarios by revisiting relatively small amounts of data.

The main limitations of this study reside on the relatively short sample size of the trials, the lack of patients treated with immunotherapy and the geographical restriction of the trials to Spain. Other pitfalls, such as the lack of complete annotation for some relevant clinical and cytogenetic variables in all trials (e.g., performance status and chromosome 1 abnormalities), suggest the existence of significant room for improvement. Future advances in MM prognostication should move in these directions.

In conclusion, the present work describes a new, simple and easy to use prognostic model in newly diagnosed MM whose predictions are independent of ISS and R-ISS scores. Notably, the model is particularly well suited in order to classify R-ISS score 2 patients in 2 subgroups with significantly different survival. The reproduction of this clusterization in different MM databases developed by other national and international working groups is recommended, and their associations with drug response in clinical trials should be studied. The combination of ISS, R-ISS and unsupervised machine learning clusterization is a promising approximation in order to improve MM risk stratification.

## Supplementary information

Supplementary Tables and Figures
