# Supplementary material for: Unsupervised machine learning improves risk stratification in newly diagnosed multiple myeloma: an analysis of the Spanish Myeloma Group
Source: Blood Cancer J. 2022 Apr 25;12(4):76. doi: 10.1038/s41408-022-00647-z (PMC9038663; doi:10.1038/s41408-022-00647-z)
Supplement: Supplementary file 1 — Supplementary Tables and Figures [file 41408_2022_647_MOESM1_ESM.pdf]

Supplementary Figure 1. Results of GMM-EM-based clusterization with 3 and 4 optimal clusters across the 3 trial cohorts.

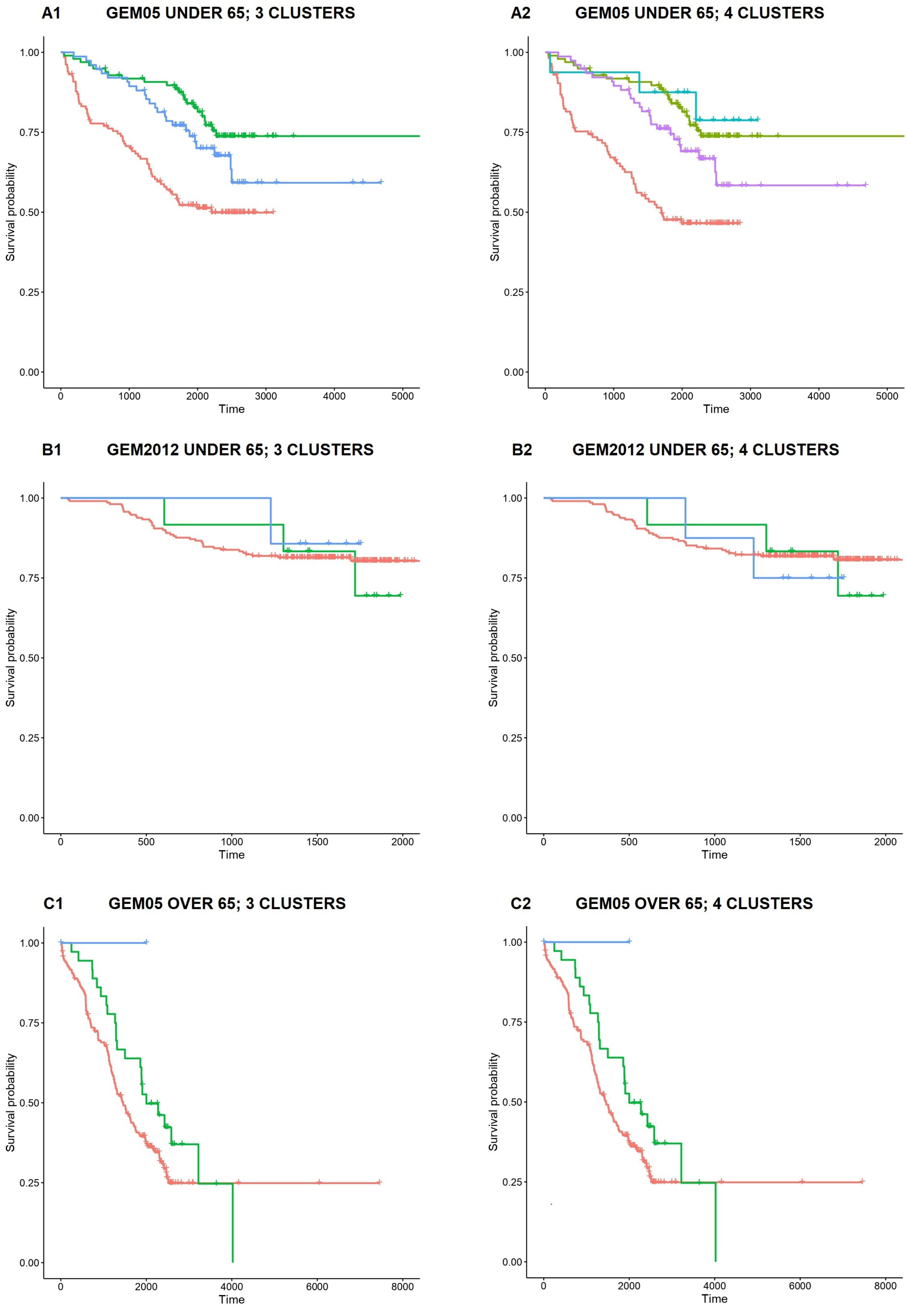

Supplementary Figure 2. Kaplan-Meier plots representing the overall survival of R-ISS I, II and III subgroups in the GEM05 under 65 years (A-C), GEM05 over 65 years (D-F) and GEM2012 under 65 years (G-H) trials. The red line represents patients in the "high-risk" cluster (cluster I), and the blue line represents patients in the "low-risk" cluster (cluster II).

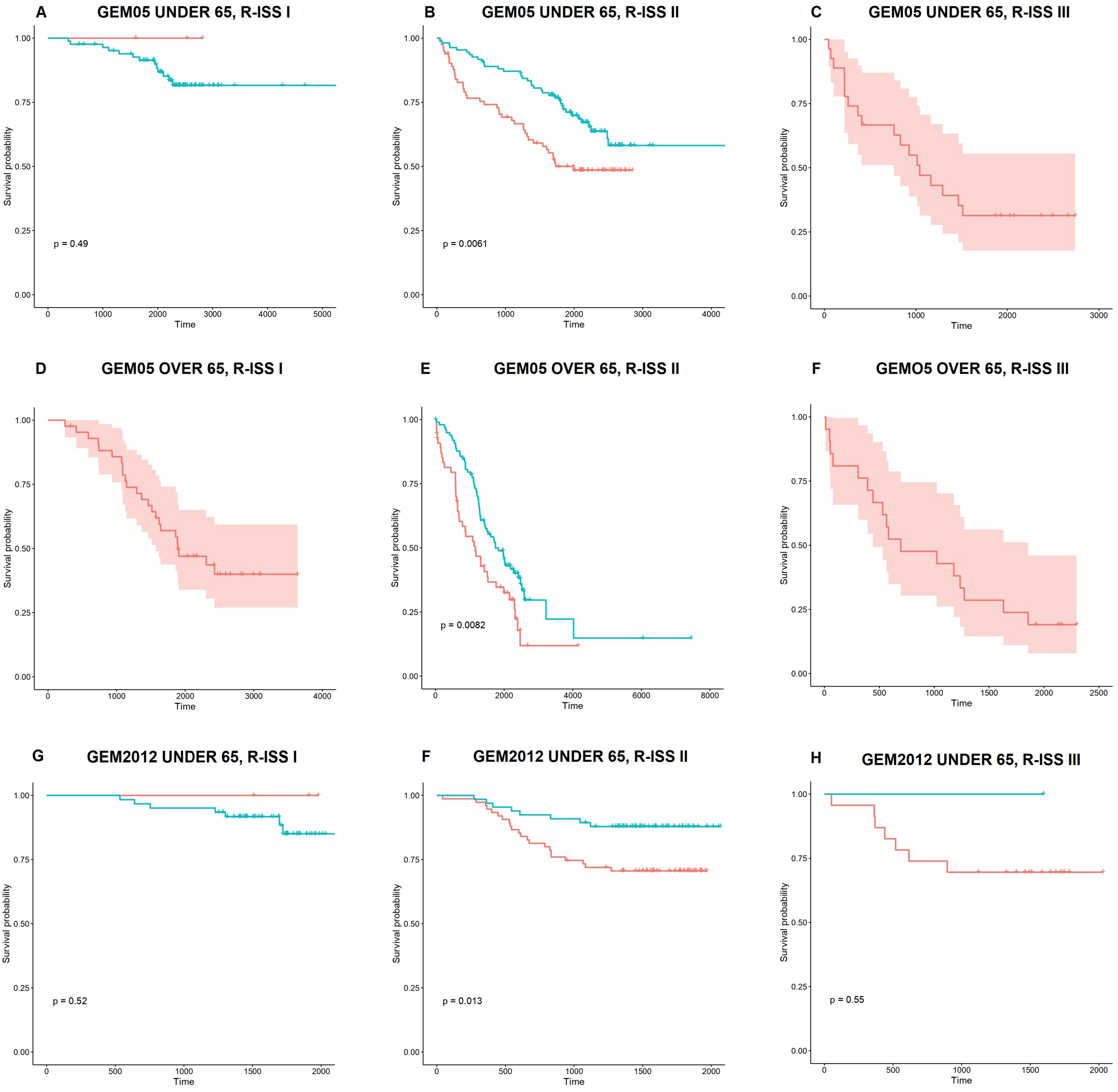

Supplementary Figure 3. Kaplan-Meier plots representing the overall survival of all patients as well as that of R-ISS I, II and III subgroups in the GEM05 under 65 years (A-D), GEM05 over 65 years (E-H) AND GEM2012 under 65 years (I-L) trials. The red line represents patients in the "high-risk" cluster (cluster I), and the blue line represents patients in the "low-risk" cluster (cluster II).

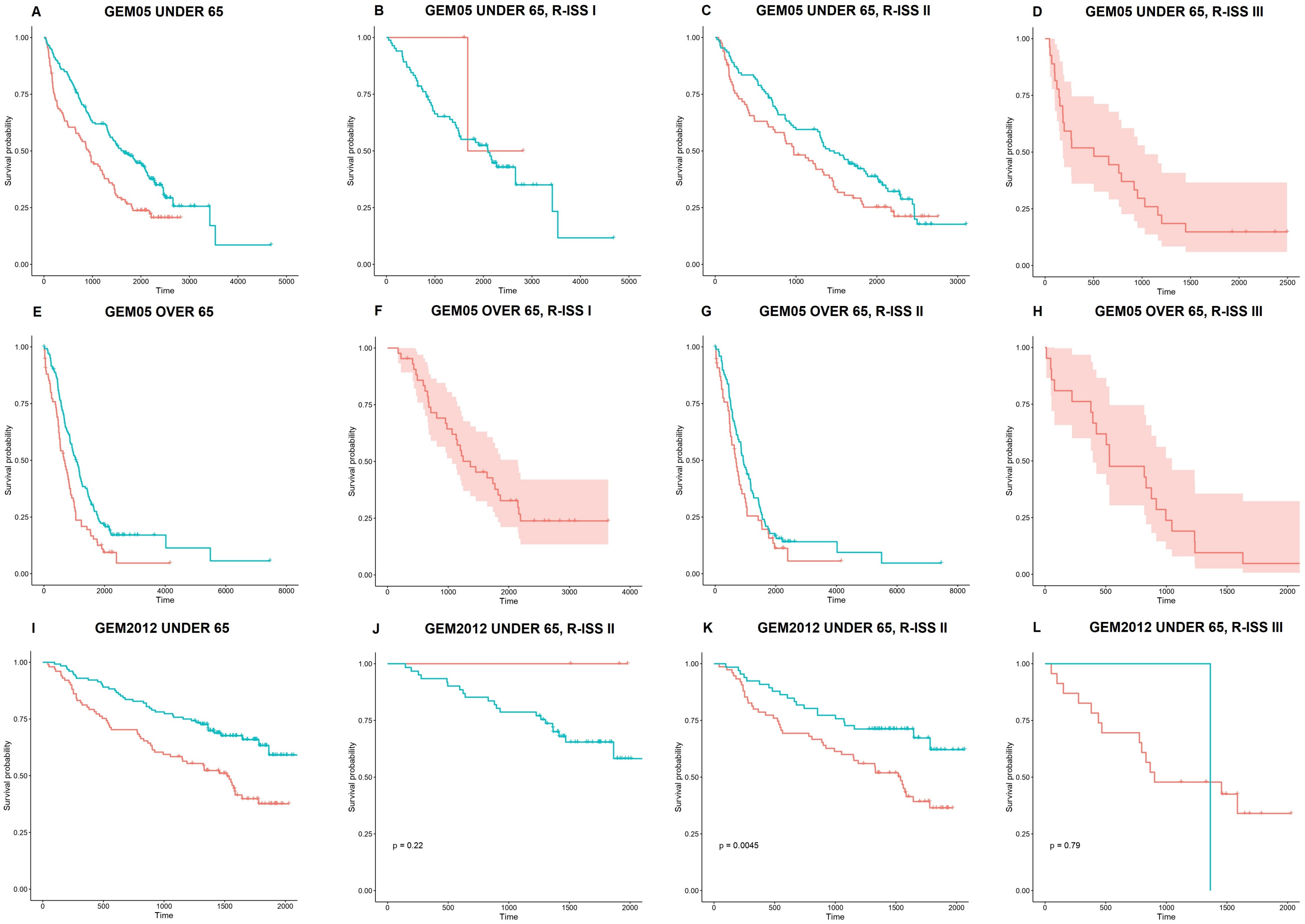

Supplementary Figure 4. Kaplan-Meier plots representing the overall survival of R-ISS I, II-low risk, II-high risk and III subgroups in the GEM05 under 65 years (A), GEM2012 under 65 years (B) and GEM05 over 65 years (C) trials.

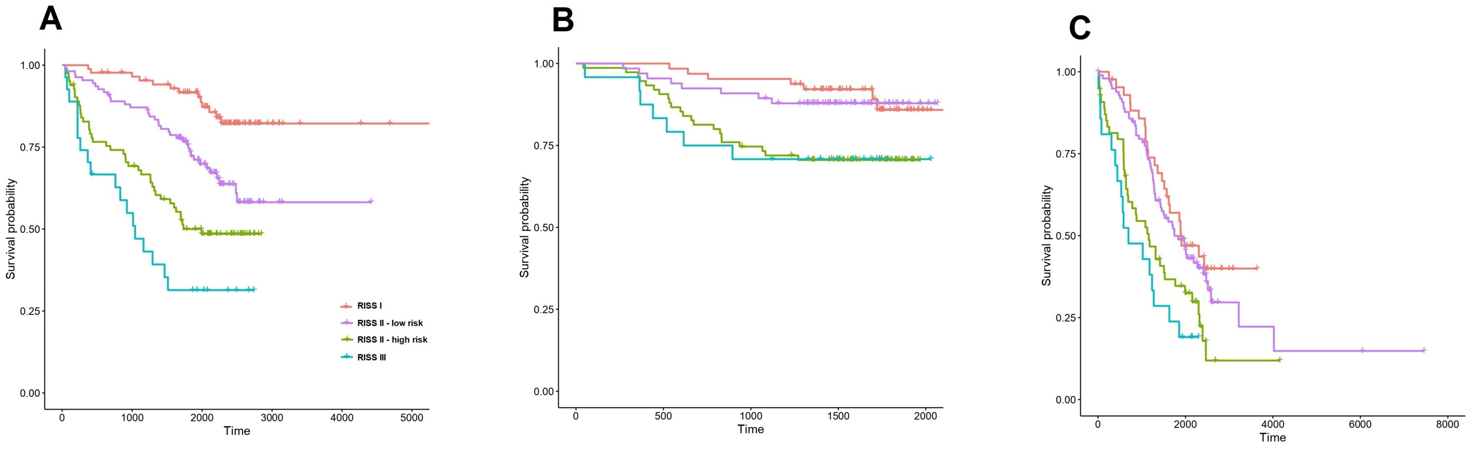

Supplementary Figure 5. Time-dependent AUCs representing the precision of the different models to predict overall survival.

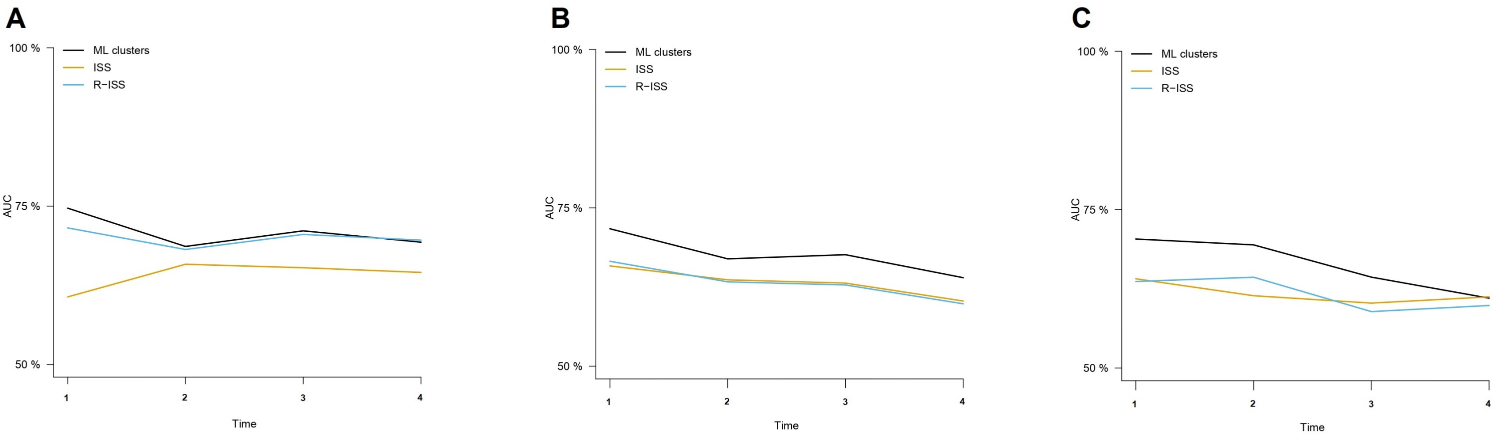

	GEM05 under 65	GEM05 over 65	GEM2012 under 65
*Hemoglobin**	p-val 1.02 x 10-5 [-1.4, -0.60]	p-val 2.82 x 10-5 [-1.40, -0.50]	p-val 4.98 x 10-4 [-1.60, -0.30]
*Albumin-adjusted calcium**	p-val 0.84 [-0.26, 0.30]	p-val 0.44 [-0.20, 0.46]	p-val 0.24 [-0.30, 0.09]
*B2-microglobulin**	p-val 8.45 x 10-5 [0.45, 1.37]	p-val 5.15 x 10-7 [1.01, 2.32]	p-val 2.07 x 10-4 [0.40, 1.47]
*High risk cytogenetics***	p-val < 1 x 10-5	p-val < 1 x 10-5	p-val < 1 x 10-5
*t(4;14)***	p-val < 1 x 10-5	p-val < 1 x 10-5	p-val < 1 x 10-5
*t(14;16)***	p-val < 1 x 10-5	p-val 0.12	p-val < 1 x 10-5
*17p del***	p-val < 1 x 10-5	p-val < 1 x 10-5	p-val < 1 x 10-5
*Raised LDH***	p-val < 1 x 10-5	p-val < 1 x 10-5	p-val < 1 x 10-5
*Durie-Salmon stages I, II and III****	Cluster 1: 3.30%, 77.05%, 50.41%<br>Cluster 2: 7.78%, 52.85% & 39.38%	Cluster 1: 7.89%, 38.16% & 53.94%<br>Cluster 2: 7.04%, 59.15% & 33.80%	Cluster 1: 12.87%, 33.66% & 53.47%<br>Cluster 2: 8.59%, 42.19% & 49.22%

* Wilcoxon Rank-Sum Test
plus 95% Confidence Interval

**Fisher's Test

***Descriptive

Supplementary Table 1.
Differential distribution between the 2 unsupervised clusters of the different predictive variables which compose it. Continuous variables were analyzed with the Wilcoxon Rank-Sum Test and binomial variables were analyzed with Fisher's test. For Durie-Salmon stage distribution, descriptive statistics are provided.

	GEM05 under 65		GEM05 over 65		GEM2012 under 65	
Variables	***p-val***	***HR (95%CI)***	***p-val***	***HR (95%CI)***	***p-val***	***HR (95%CI)***
II-cluster 2 vs I	2.00 x 10-3	2.71 [1.44, 5.11]	0.42	1.21 [0.75, 1.95]	0.68	1.23 [0.45, 3.42]
III vs II-cluster 1	0.06	1.69 [0.97, 2.95]	0.21	0.96 [0.39, 1.23]	0.90	0.95 [0.40, 2.22]

Supplementary Table 2. Cox regression results comparing the outcomes of R-ISS I vs II-low risk and R-ISS III vs II-high risk.

	**GEM05 under 65**	**GEM05 over 65**	**GEM2012 under 65**
N	191	154	141
Cluster I vs II	42.93% vs 57.07%	35.71% vs 64.29%	41.13% vs 58.87%
High Risk Cytogenetics Cluster I vs II	52.43% vs 0%	47.27% vs 0%	56.63% vs 0%
Durie-Salmon stages: I, II & III	Cluster 1: 3.66%, 46.34%, 50.00%<br>Cluster 2: 6.42%, 46.79%, 46.79%	Cluster 1: 10.91%, 41.82%, 47.27%<br>Cluster 2: 6.06%, 51.52%, 42.42%	Cluster 1: 9.64%, 33.73%, 56.63%<br>Cluster 2: 5.17%, 41.38%, 53.45%
Median Hemoglobin (g/dL)	10.5 *vs* 10.3	9.9 *vs* 10.2	10.4 *vs* 11.0
Median albumin-adjusted calcium (mg/dL)	9.73 *vs* 9.88	9.88 *vs* 10.18	9.5 *vs* 9.6
Median B2-microglobulin (mg/dL)	3.35 *vs* 4.09	4.3 *vs* 4.3	3.5 *vs* 4.36
Raised LDH	41.46% *vs* 0%	27.27% *vs* 0%	27.71% *vs* 0%
Raised LDH or high risk cytogenetics	86.59% *vs* 0%	69.09% *vs* 0%	72.29% *vs* 0%
Survival of Cluster I patients with and without either high risk cytogenetics or raised LDH	HR 1.12, p-value 0.81	HR 1.33, p-value 0.41	HR 0.98, p-value 0.96

Supplementary Table 3.
Distribution of the variables included in the unsupervised machine learning model according to cluster membership for R-ISS 2 patients.
Also represented are the results of the survival analysis between “high-risk” cluster I patients with and without either high risk cytogenetics or raised LDH.

	GEM05 under 65	GEM2012 under 65	GEM05 over 65
ISS	0. 623	0. 598	0. 578
RISS	0. 648	0. 600	0. 563
UNSUPERVISED MODEL	0. 645	0. 653	0. 591
ISS+RISS	0. 615	0. 592	0. 577
ISS + UNSUPERVISED MODEL	0. 690	0. 659	0. 619
RISS + UNSPERVISED MODEL	0. 688	0. 641	0. 610
ISS + RISS + UNSUPERVISED MODEL	0. 700	0. 644	0. 615

Supplementary Table 4.
C-indexes obtained after 10-fold cross-validation of the different cox regression models.
